# A Conditional Mutual Information-Based Approach for Robust Multi-Source Feature Selection in IoT Systems

**DOI:** 10.3390/s26082340

**Published:** 2026-04-10

**Authors:** Hao Jiang, Shenjie Xu, Yong Shen

**Affiliations:** School of Computer Science, Jiangsu University of Science and Technology, Zhenjiang 212003, China; 231210701109@stu.just.edu.cn (H.J.); 231210704108@jsust.edu.cn (S.X.)

**Keywords:** feature selection, multi-source features, conditional information, residual modeling, dimensionality reduction

## Abstract

Feature selection is essential for high-dimensional multi-source feature analysis, particularly in Internet of Things (IoT) environments characterized by data heterogeneity, redundancy, and noise. To address the need to balance classification performance, dimensionality reduction, and selection stability, this study proposes a residual-based conditional mutual information and feedback fusion (RCMF) feature-selection method. Inspired by the idea of conditional mutual information, the proposed method first introduces a residual-based indicator to characterize the incremental discriminative information retained by a candidate feature under given conditional constraints. On this basis, model-driven predictive contribution and stability score are further incorporated, and the weights of different evaluation components are iteratively updated during the feature-selection process to achieve adaptive fusion. In this way, the method jointly considers conditional discriminative information, task relevance, and selection consistency within a unified feature-evaluation procedure. Experiments on multiple publicly available benchmark and IoT-related datasets validate the rationality and effectiveness of the proposed method.

## 1. Introduction

With the continued development of the Internet of Things (IoT), intelligent communication, and sensing technologies, device operations and network interactions generate large volumes of data from diverse sources and with heterogeneous structures [1]. These features provide a rich informational basis for device identification, behavior modeling, and security authentication. However, in IoT scenarios, multi-source features are typically characterized by strong heterogeneity, high dimensionality, and complex noise distributions, while redundancies and mutual interference may exist among features from different sources [2]. Against this backdrop, effectively selecting key features with strong discriminative power and good stability from complex feature sets has become a critical factor for enhancing model performance.

Feature selection, as a fundamental technique in high-dimensional data analysis, is widely employed for information compression and feature optimization. Classical approaches can be categorized into filter, wrapper, and embedded methods [3]. Filter methods rank features based on statistical measures, offering high computational efficiency but often failing to capture nonlinear relationships or interactions among features [4], which may result in retaining redundant information. To improve feature-selection quality, Jiang et al. [5] proposed an unsupervised method based on incremental forward iterative Laplacian scores, which enhances discriminative capability by incorporating feature importance; however, its reliance on K-nearest neighbor structures may introduce redundant features. Salmi et al. [6] constructed a feature scoring mechanism based on similarity constraints, recalculating the similarity matrix in a subspace to better capture feature correlations, yet the additional matrix computation increases complexity. Fejzic et al. [7] combined correlation analysis with statistical significance testing to optimize the selection process, which improved classification performance but exhibited strong dependence on data distribution assumptions and incurred additional overhead due to the multi-stage testing procedure.

Wrapper methods evaluate subsets of features through model performance, enabling the capture of complex combinatorial relationships, but they require repeated model training and are computationally expensive, while the search process often lacks interpretability [8]. Yilmaz et al. [9] applied an improved ant colony optimization algorithm for feature selection, assessing feature importance through path frequency ranking; although effective for high-dimensional data, the approach is sensitive to parameter settings. Hu et al. [10] proposed a particle swarm optimization-based federated feature-selection framework, achieving multi-party collaborative optimization under privacy-preserving conditions, but performance is influenced by communication overhead and data distribution heterogeneity. Pan et al. [11] enhanced the grey wolf optimization algorithm by introducing competition-guided strategies and differential evolution mechanisms to improve global search capability, though a trade-off between computational complexity and convergence speed remains. Li et al. [12] combined artificial fish swarm algorithms with population mutation mechanisms, achieving high accuracy across multiple datasets, albeit at the cost of a relatively time-consuming overall optimization process.

Embedded methods integrate feature selection into the model training stage, offering advantages in efficiency and correlation modeling. However, these methods are typically dependent on specific model structures and cannot operate independently of the underlying model. Furthermore, most importance-based interpretability techniques only provide feature ranking results, lacking systematic search mechanisms for feature subset combinations. Hao et al. [13] proposed a multi-view multi-label embedded feature-selection model that optimizes the weights among different feature perspectives within a unified framework, but its stability may be affected in heterogeneous structures or in the presence of label noise. Zhao et al. [14] improved search capability through modified seeding strategies and chaotic population mechanisms, yet algorithmic complexity correspondingly increased. Wang et al. [15] quantified feature contributions using a game-theoretic model, offering interpretability advantages, but with high computational costs and primarily focusing on ranking rather than combinatorial optimization.

In summary, existing feature-selection methods still face challenges in multi-source feature scenarios. On one hand, techniques emphasizing interpretability often lack global combinatorial search capability; on the other hand, algorithms with strong search power are usually computationally intensive and exhibit limited transparency in decision-making. In addition, most approaches rely on single statistical metrics or importance evaluations from a single model [16]. In multi-source feature environments, the relationships between features and labels often depend on interactions among information from different sources, making single-source modeling insufficient to capture complex dependency structures [17]. Moreover, such approaches are susceptible to noise and distributional differences, resulting in unstable feature-selection outcomes.

Therefore, it is necessary to develop a feature-selection method that can effectively model dependencies among multi-source features, balance discriminative power and stability, and support combinatorial optimization, thereby improving the reliability and generalizability of feature selection in complex environments.

To address the aforementioned challenges, this paper proposes a feature-selection algorithm for high-dimensional multi-source IoT data based on residual-approximate conditional mutual information and a feedback fusion mechanism. The proposed method integrates conditional dependency modeling, model-driven importance analysis, and a bootstrap-based stability evaluation strategy during feature assessment, while employing iterative feedback to dynamically update feature weights. By modeling the incremental information contributed by each feature under the condition of other features, the algorithm is capable of identifying key features with discriminative value in cross-feature contexts. Meanwhile, it improves selection consistency under data perturbations and noisy environments, thereby enhancing the robustness and reliability of feature selection in complex multi-source scenarios.

The main contributions of this paper are summarized as follows:

1. To address the difficulty of modeling complex dependencies among features in high-dimensional multi-source environments, a feature-evaluation method based on residual-approximate conditional information is proposed. By employing residual modeling, conditional dependencies are transformed into a residual-based correlation analysis, enabling the characterization of the incremental discriminative information of features under the constraints of conditional features. This approach improves both the effectiveness and stability of feature evaluation while maintaining computational efficiency.
2. A multi-metric feature-evaluation framework is developed by integrating model-driven predictive contribution and bootstrap-based stability assessment. An iterative feedback strategy based on the current feature subset is further introduced to dynamically update feature weights. This mechanism evaluates features from three perspectives—discriminative capability, task relevance, and stability—allowing the feature-selection process to be progressively optimized while enhancing the consistency of the results.
3. Extensive experiments, including comparative studies and ablation analyses, are conducted on multiple public datasets to evaluate the proposed method in terms of average classification performance, feature compression effectiveness, and stability. The results demonstrate that the proposed method achieves superior or competitive performance on most datasets, while effectively reducing the number of selected features without compromising classification accuracy. Ablation and replacement experiments further verify the contribution and synergistic effects of the individual components.

## 2. Materials and Methods

### 2.1. Theoretical Foundations

#### 2.1.1. Conditional Mutual Information

Conditional mutual information (CMI) measures the dependency between a feature and the target label given other variables. It quantifies the additional information contributed by a feature when certain conditional information is already known, thereby evaluating its conditional discriminative capability [18].

From an information-theoretic perspective, CMI captures general statistical dependencies beyond simple linear correlations. For this reason, it is widely adopted in feature selection to assess the incremental contribution of a feature under specified conditions.

The formulation of conditional mutual information is given as follows:

(1) $$CMI(X_{i};Y|Z)=\sum_{x_{i}}\sum_{y}\sum_{z}p(x_{i},y,z)\log\frac{p(x_{i},y|z)}{p(x_{i}|z)p(y|z)}$$

In the above formulation, $p(x_{i},y,z)$ denotes the joint probability distribution of feature $X_{i}$, label Y, and the conditional feature set Z. The term $p(x_{i}|z)$ represents the conditional probability that feature $X_{i}$ takes a specific value given Z; p(y|z) denotes the conditional probability of label Y under the same condition. The term $p(x_{i},y|z)$ corresponds to the joint conditional probability that $X_{i}$ and Y simultaneously take specific values given Z.

The formulation indicates that a larger value of conditional mutual information implies that the feature provides greater independent information when conditioned on other features, thereby demonstrating stronger discriminative capability for the classification task.

#### 2.1.2. Predictive Contribution

Predictive contribution is used to quantify the extent to which a feature contributes to the model output in a practical classification task. In wireless device recognition, different features may have varying levels of importance with respect to the classification results. By training a nonlinear classifier, the role of each feature within the model can be directly evaluated, thereby providing a more accurate reflection of its discriminative capability.

In this study, the XGBoost classifier [19] is employed to train the current feature set, and the cumulative gain of each feature at tree-splitting nodes is calculated. The predictive contribution is defined as follows:

(2) $$C(X_{i})=\sum_{t\in T}\sum_{n\in N_{t}}Gain(X_{i},n)$$

In the above formulation, T denotes the set of trained decision trees, $N_{t}$ represents the set of all nodes in the t-th tree, and $Gain(X_{i},n)$ indicates the gain contributed by feature $X_{i}$ when used for splitting at node n.

The formulation indicates that a higher predictive contribution value implies that the feature provides more information to the classifier and contributes more significantly to the prediction results. Unlike purely statistical methods, predictive contribution can capture nonlinear relationships between features and labels, making feature evaluation more aligned with practical task requirements.

#### 2.1.3. Feature-Selection Stability

Feature-selection stability measures the consistency with which a feature is selected under data perturbations or sample variations. A feature with high stability indicates that it maintains discriminative capability across different data subsets, thereby ensuring the reliability and generalization ability of the final feature subset [20]. For wireless device recognition tasks, the stability metric helps mitigate instability in feature selection caused by small-sample noise or randomness, thus improving the applicability of the model in real-world environments.

In this study, multiple data subsets are generated using bootstrap sampling. Feature scoring is performed on each subset, and the top K features are selected accordingly. The stability is defined as follows:

(3) $$S(X_{i})=\frac{1}{B}\sum_{b=1}^{B}1\left\{X_{i}\in S_{b}\right\}$$

In the above expression, B denotes the number of sampling iterations, $S_{b}$ represents the selected feature set obtained from the b-th subset, and $1\left\{X_{i}\in S_{b}\right\}$ is the indicator function, which equals 1 if the feature is selected and 0 otherwise.

The formulation indicates that a higher stability value implies a greater frequency of repeated selection across different subsets, thereby reflecting more reliable discriminative capability. By incorporating the stability score, the algorithm considers both discriminative power and robustness in the overall evaluation, leading to a more stable feature subset.

### 2.2. RCMF Algorithm

To effectively quantify the independent contribution of candidate features to classification tasks under high-dimensional settings, given the presence of other features, this paper proposes a Residual-based Conditional Discriminative Measure (RCDM). This measure is designed to capture the discriminative information retained by a candidate feature under the constraints of the current conditional feature set.

The proposed method is inspired by the concept of conditional mutual information, which focuses on the relationship between a candidate feature and the target variable conditioned on other features. Specifically, a regression model is employed to reduce the explainable component of the candidate feature with respect to the conditional features, decomposing it into an explainable component and a residual component. In this way, the complex conditional dependency is transformed into a correlation analysis problem at the residual level. The mutual information between the residual and the target label is then computed to evaluate the discriminative capability of the candidate feature under the given conditions.

From the perspective of conditional dependency, this approach mitigates the influence of redundant features on the evaluation results to a certain extent and provides an effective criterion for feature selection. The proposed measure is able to reflect the additional discriminative information contributed by a candidate feature under the constraints of the current conditional feature set, thereby offering a practical means for assessing conditional relevance in the feature-selection process.

First, the influence of the conditional feature set on the candidate feature is removed, yielding the residual representation of the feature:

(4) $$R_{X_{i}}=X_{i}-Z{\hat{\beta }}_{i}$$

In the above expression, ${\hat{\beta }}_{i}$ denotes the coefficient vector estimated through a regression method. The resulting residual represents the independent information retained in the feature after removing the portion explained by the conditional variables.

Similarly, the label Y is processed under the same conditional variables to obtain the corresponding residual representation of the label:

(5) $$R_{Y}=Y-Z\hat{\gamma }$$

In the above expression, $\hat{\gamma }$ represents the estimated regression coefficients.

On this basis, the Residual-based Conditional Discriminative Measure is defined as follows:

(6) $$RCDM(X_{i})=I(R_{X_{i}};R_{Y})$$

In the above expression, $I(R_{X_{i}};R_{Y})$ denotes mutual information. A larger value indicates that, after removing the explainable component associated with the conditional features, the candidate feature still provides additional discriminative information. If the value is close to zero, it suggests that the feature contributes little new information under the constraints of the current conditional feature set. Conversely, a small or near-zero value implies that the discriminative capability of the feature primarily arises from its correlation with the conditional features rather than from its own independent contribution.

In the computation of RCDM, the conditional feature set is formed by the currently selected feature subset and is progressively expanded throughout the feature-selection process. To mitigate the instability of regression models under high-dimensional conditions, the size of the final feature subset is controlled in the experiments such that the dimensionality of the conditional features remains lower than the number of samples. This ensures that the regression model operates within a relatively stable estimation regime.

After obtaining the Residual-based Conditional Discriminative Measure, a fused evaluation framework is further constructed by integrating the model-driven predictive contribution and the stability score. The residual conditional discriminative measure evaluates the independent discriminative capability of a feature under controlled conditional variables. The predictive contribution reflects the actual task-related importance of the feature within a nonlinear classification model. The stability score measures the consistency of feature selection under data perturbations. Together, these three components provide a comprehensive evaluation of features from the perspectives of conditional dependency, model contribution, and robustness.

Based on the three metrics described above, a feature-selection algorithm named Residual-based Conditional Mutual Information and Feedback Fusion (RCMF) is proposed. The algorithm integrates these indicators through a weighted fusion mechanism and constructs a comprehensive scoring function as follows:

(7) $$F(X_{i})=w_{r}RCDM(X_{i})+w_{c}C(X_{i})+w_{s}S(X_{i})$$

In the above expression, $w_{r}$, $w_{c}$, and $w_{s}$ are weight parameters used to balance the relative importance of the three indicators. By adjusting these weights, the algorithm can be adapted to different types of multimodal data and varying task requirements.

To further optimize the feature subset, a dynamically updated fusion feedback mechanism is introduced. In each iteration of the feature-selection process, the residual-based conditional discriminative measure and the predictive contribution are recalculated for the current set of candidate features. The fusion weights are then adaptively updated according to the distribution of the current scores, forming an iterative update process driven by the selected feature subset. As the feature subset is progressively expanded, both the evaluation metrics and their corresponding weights are dynamically adjusted. This mechanism enables the gradual reinforcement of features with stable and independent discriminative capability, while suppressing the influence of redundant or noisy features. During the iterative selection process, a forward selection strategy is adopted, where one optimal feature is added to the subset at each iteration. The number of iterations is determined by a predefined feature size, and the process terminates once the desired number of features is reached in the experiments.

In the multi-metric fusion process, different evaluation metrics often exhibit distinct distribution characteristics in the feature space. Some metrics produce large score variations across features, demonstrating strong discriminative ability, whereas others exhibit more concentrated score distributions, providing relatively smoother distinctions in feature importance. Therefore, it is necessary to adaptively adjust the contribution of each metric based on its distribution characteristics on the current dataset.

Based on this consideration, a variance-based weighting strategy is adopted. By measuring the dispersion of each metric across feature dimensions, its discriminative capability in feature ranking can be characterized, and the corresponding fusion weights are dynamically adjusted. When a metric exhibits large score differences among features, it indicates strong discriminative power and is thus assigned a relatively higher weight in the fusion process. Conversely, when the score distribution is more concentrated, its weight is appropriately reduced.

Let $\sigma_{r}$, $\sigma_{c}$, and $\sigma_{s}$ denote the variances of the residual conditional discriminative measure, predictive contribution, and stability score on the current feature set, respectively. The weights are defined as follows:

(8) $$w_{j}=\frac{\sigma_{j}^{2}}{\sigma_{r}^{2}+\sigma_{c}^{2}+\sigma_{s}^{2}},\;j\in \left\{r,c,s\right\}$$

Based on the above rules, the pseudocode of the RCMF algorithm is presented in Algorithm 1.
**Algorithm 1.** RCMF Workflow$\begin{array}{l} \mathrm{Input}\text{:}\;\mathrm{Original}\;\mathrm{feature}\;X=\left\{X_{1},X_{2},\cdots ,X_{n}\right\},\mathrm{label}\;Y,\mathrm{target}\;\mathrm{number}\;\mathrm{of}\;\mathrm{selected}\;\mathrm{features}\;K\\ \mathrm{Output}\text{:}\;\mathrm{Optimal}\;\mathrm{feature}\;\mathrm{subset}\;S_{RCMF}\\ 1\;S_{RCMF}=Ø\\ 2\;S_{temp}=Ø\\ 3\;X_{max}=0\\ 4\;\mathrm{Precompute}\;\mathrm{stability}\;\mathrm{score}\;S(X_{i})\\ 5\;\mathrm{for}\;i=1\;\mathrm{to}\;K\;\mathrm{do}\\ 6\;\;\;\mathrm{for}\;\mathrm{each}\;X_{j}\in X\;\mathrm{do}\\ 7\;\;\;\;\;\mathrm{Calculate}\;\mathrm{residual}\;\mathrm{conditional}\;\mathrm{discriminant}\;\mathrm{measure}\;RCDM(X_{j})\\ 8\;\;\;\;\;\mathrm{Calculate}\;\mathrm{predictive}\;\mathrm{contribution}\;C(X_{j})\\ 9\;\;\;\;\;\mathrm{Obtain}\;\mathrm{precomputed}\;\mathrm{stability}\;\mathrm{score}\;S(X_{j})\\ 10\;\;\mathrm{end}\;\mathrm{for}\\ 11\;\;\mathrm{Calculate}\;\mathrm{fusion}\;\mathrm{weights}\;w_{r},\;w_{c},\;w_{s}\\ 12\;\;\mathrm{for}\;\mathrm{each}\;X_{j}\in X\;\mathrm{do}\\ 13\;\;\;\;\mathrm{Compute}\;\mathrm{fusion}\;\mathrm{score}\;F(X_{j})\\ 14\;\;\;\;\;S_{temp}=S_{temp}+\left\{F_{X_{j}}\right\}\;\\ 15\;\;\mathrm{end}\;\mathrm{for}\\ 16\;\;X_{max}=\arg max\left(S_{temp}\right)\\ 17\;\;S_{RCMF}=S_{RCMF}+\left\{X_{max}\right\}\\ 18\;\;X=X-\left\{X_{max}\right\}\\ 19\;\;S_{temp}=Ø\\ 20\;\;\;\mathrm{end}\;\mathrm{for}\\ 21\;\;\;\mathrm{return}\;S_{RCMF} \end{array}$

Compared with fixed-weight strategies, the proposed approach can adaptively adjust to the distribution characteristics of evaluation metrics across different datasets. Fixed weights typically rely on manual settings and may fail to balance the contributions of different metrics under varying data conditions. In contrast, the variance-based weighting scheme reflects the discriminative differences in metrics on the current dataset, thereby improving the adaptability of the fusion evaluation. Moreover, compared with learning-based weighting methods, the proposed strategy does not require additional parameter learning or optimization objectives, avoiding increased model complexity and potential overfitting. While maintaining computational efficiency, it also offers good interpretability. Furthermore, in feature-selection tasks, the role of evaluation metrics primarily lies in their ability to distinguish feature importance rankings, and variance effectively captures the degree of differentiation in feature scores. Therefore, the variance-based weighting scheme enables adaptive fusion of multi-metric information without introducing additional computational burden, demonstrating strong practical applicability.

### 2.3. Complexity Analysis of the RCMF Algorithm

Considering the computational procedures of each module in the RCMF algorithm, the time complexity of each component can be analyzed individually. The overall computational complexity of the RCMF algorithm is then obtained by aggregating the complexities of all modules.

- Computational Complexity of Residual Scoring

The residual score is computed by fitting a linear regression model for each feature and then calculating the mutual information between the residuals and the label to evaluate feature importance.

Assume there are n samples and m features. For each feature, a linear regression model is fitted with a time complexity of O(n⋅m). The mutual information computation between the residual and the label has a time complexity of O(n). Therefore, the total complexity for each feature is O(n⋅m). For all m features, the overall computational complexity is $O(n\cdot m^{2})$.

The computational complexity of a single selection round is $O(n\cdot m^{2})$. Since the algorithm adopts a stepwise iterative feature-selection strategy with a total of  K selection rounds, the overall computational complexity of the residual-based conditional discriminative measure component can be expressed as  $O(K\cdot n\cdot m^{2})$.

- Computational Complexity of Predictive Contribution

The predictive contribution is computed by training an XGBoost model and evaluating the contribution of each feature to the prediction results. The training complexity of XGBoost is approximately O(n⋅m⋅logm), where n denotes the number of samples, m represents the number of features. Therefore, the overall computational complexity for calculating predictive contribution using the full feature set is O(n⋅m⋅logm). After K iterations, the overall complexity of the predictive contribution component can be approximated as O(K⋅n⋅m⋅logm).

- Computational Complexity of Stability Scoring

The stability score is computed based on bootstrap sampling to estimate the frequency with which each feature is selected. Assume that B bootstrap iterations are performed, and each bootstrap sample contains n samples. If the time complexity of computing mutual information for each evaluation is O(n⋅m), then the total computational complexity of the stability scoring process is O(B⋅n⋅m).

Based on the above analysis, the overall computational complexity of the RCMF algorithm is the sum of the complexities of the three components, namely residual scoring, predictive contribution, and stability scoring. Since the term $K\cdot n\cdot m^{2}$ is the dominant term among them, the overall computational complexity of the RCMF algorithm can be expressed as: $O(K\cdot n\cdot m^{2})$. In practical applications, the number of selected features K is usually much smaller than the total number of original features m; therefore, despite the iterative nature of the algorithm, the overall computational complexity remains acceptable.

## 3. Results

Accuracy and stability are two important metrics for evaluating the performance of a feature-selection algorithm. A feature-selection method is considered to have higher accuracy if it achieves a higher recognition rate while selecting a smaller number of features [21]. Stability refers to the similarity of the selected feature subsets across different cross-validation folds under the condition that the number of selected features remains the same. A higher similarity indicates stronger stability of the feature-selection algorithm.

In the following experiments, the proposed RCMF algorithm is compared with four feature-selection methods: interaction weight-based feature selection (IWFS) [22], Random Forest (RF) [23], Recursive Feature Elimination with Cross-Validation (RFECV) [24], and conditional relevance feature selection (CRFS) [25]. The comparison is conducted on different types of datasets in terms of three aspects: classification accuracy, the number of features in the selected subset, and algorithm stability. All experiments were implemented in Python (Version 3.10) using Scikit-learn (Version 1.5.1) and XGBoost (Version 1.7.6).

### 3.1. Dataset Description

The datasets used in this chapter are sourced from the University of California, Irvine (UCI) Machine Learning Repository http://archive.ics.uci.edu/ml/index.php (accessed on 10 August 2025) and publicly available datasets from Kaggle https://www.kaggle.com/datasets (accessed on 20 August 2025). The datasets include both IoT and non-IoT datasets. Detailed information about the datasets is provided in Table 1.

In Table 1, the datasets are listed in order of their indices as follows: Zoo [26], Car Evaluation [27], Seeds [28], Raisin [29], IoT-23 Cleaned [30], TON_IoT [31], RT-IoT2022 [32], and IoT Device Identification [33]. The first four are classical benchmark datasets, while the latter four are IoT-related datasets.

### 3.2. Data Preprocessing

To avoid the influence of different feature scales on the evaluation results, the raw data are normalized so that all values are mapped into the range [0, 1]. The normalization formula is given as follows:

(9) $$r_{i,j}=\frac{x_{i,j}-min\left(x_{j}\right)}{max\left(x_{j}\right)-min\left(x_{j}\right)}$$

In this experiment, all samples consist of numerical data. Therefore, feature values are first converted into numerical form during preprocessing. For datasets containing missing values, the mean value of the corresponding attribute is used for imputation.

### 3.3. Classification Performance Evaluation of the RCMF Algorithm

Classification performance is an important metric for evaluating feature-selection algorithms. In this section, the classification accuracies of different algorithms are compared. A higher accuracy value indicates better classification performance. The classification accuracy is calculated as follows:

(10) $$Accuracy=\frac{n}{N}\times 100\%$$

In the above expression, n denotes the number of correctly classified samples, and N represents the total number of samples.

**Figure 1:**
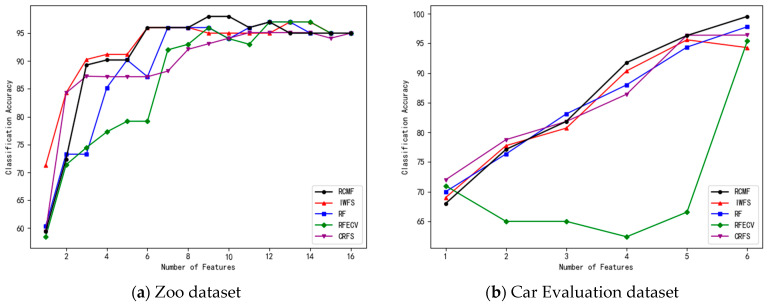
The number of different features on the Zoo and Car Evaluation datasets and their corresponding accuracy.

**Figure 2:**
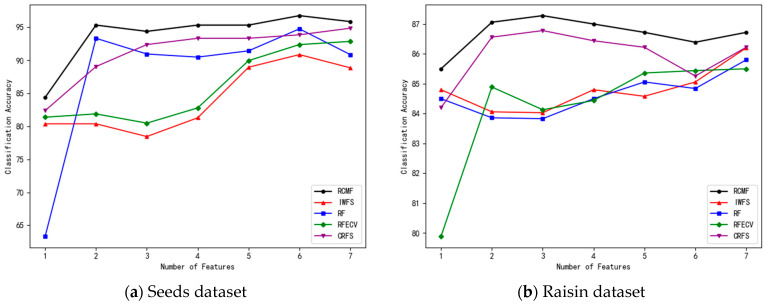
The number of different features on the Seeds and Raisin datasets and their corresponding accuracy.

**Figure 3:**
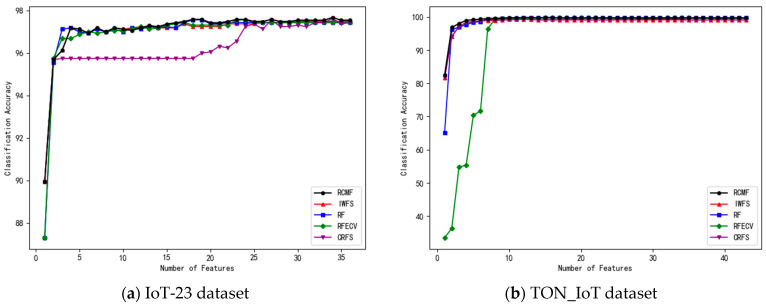
The number of different features on the IoT-23 and TON_IoT Cleaned datasets and their corresponding accuracy.

**Figure 4:**
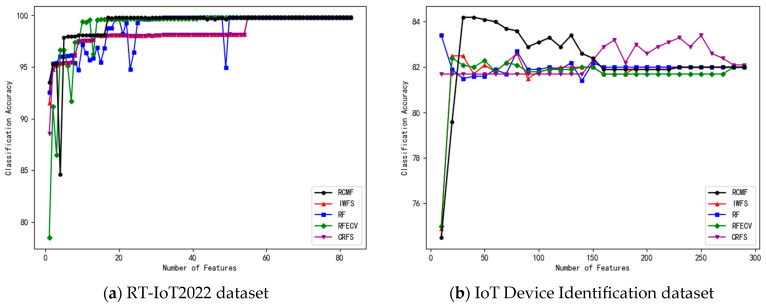
The number of different features on the RT-IoT2022 and IoT Device Identification datasets and their corresponding accuracy.

In the experiments, features are ranked in descending order according to their importance. The number of features in the feature subset is gradually increased, and 10-fold cross-validation is applied to each subset. The number of selected features and the corresponding classification accuracy of the five algorithms on the eight datasets are shown in Figure 1, Figure 2, Figure 3 and Figure 4.

Table 2 presents the highest classification accuracy (mean ± standard deviation) of IWFS, RF, RFECV, CRFS, and RCMF on different datasets based on 10-fold cross-validation. All reported results correspond to the performance of each method at its optimal feature subset size.

As shown in Table 2, RCMF demonstrates superior classification performance on most datasets. For instance, on the Zoo dataset, RCMF achieves an accuracy of 98.03%, which is significantly higher than that of IWFS and other competing methods. On the Car Evaluation dataset, RCMF attains 99.54%, showing a clear improvement over RF, RFECV, and related approaches. Similarly, on the Seeds dataset, RCMF achieves 96.76%, maintaining stable and competitive performance.

On datasets such as TON_IoT and RT-IoT2022, all methods achieve relatively high classification accuracy, approaching performance saturation. Nevertheless, RCMF still obtains the best or near-best results. Moreover, it can be observed that RCMF exhibits relatively smaller standard deviations on these datasets, indicating better stability under different data partitions.

For more challenging datasets, such as Raisin and IoT Device Identification, the overall classification performance of all methods decreases. However, RCMF still achieves the highest accuracies of 87.28% and 84.20%, respectively, demonstrating its discriminative advantage in complex scenarios.

In addition, from the perspective of standard deviation, RCMF shows smaller or comparable variation across most datasets, suggesting that it maintains stable performance while improving classification accuracy.

In summary, RCMF exhibits strong discriminative capability and stability across multiple datasets. Its advantages are reflected not only in higher classification accuracy but also in reduced performance variance under different data splits, thereby validating the effectiveness and generalization ability of the proposed feature-selection method.

In terms of the number of selected features, feature-selection algorithms aim to minimize the number of features while maintaining high classification accuracy. Table 3 presents the number of features selected by the proposed RCMF algorithm and the other four comparison methods when achieving their highest classification accuracy.

According to the results in Table 3, all five feature-selection algorithms are able to reduce the dimensionality of the original feature space to varying degrees. Overall, the proposed RCMF algorithm selects a relatively small number of features on most datasets. On the Zoo dataset, RCMF selects only 9 features, which is fewer than those selected by IWFS, RF, and RFECV. On the Raisin dataset, RCMF and CRFS both select 3 features, achieving the most significant compression effect. On the TON_IoT dataset, RCMF selects 13 features, which is the same as IWFS but substantially fewer than the 32 features selected by CRFS.

Although RCMF is not always the method with the smallest number of selected features—for example, on the IoT-23 Cleaned and RT-IoT2022 datasets, it selects 34 and 57 features, respectively—it still maintains good dimensionality reduction performance overall. In particular, on the high-dimensional IoT Device Identification dataset, RCMF reduces the feature dimension to 30, which is significantly better than IWFS and CRFS, demonstrating strong dimensionality reduction capability.

In terms of the average number of selected features, RCMF achieves a mean value of 19.75, which is considerably lower than that of IWFS and CRFS, and also lower than the average number of original features. Overall, it is comparable to RF and RFECV. In summary, RCMF achieves effective feature compression while maintaining classification performance, demonstrating its advantage in feature selection.

### 3.4. Stability Analysis of the RCMF Algorithm

The previous experiments evaluated the classification performance of the RCMF algorithm. However, for a feature-selection algorithm, stability is also a critical criterion. This subsection compares the average Kuncheva index of RCMF and other algorithms under different numbers of selected features [34]. The Kuncheva index measures the similarity between two feature subsets. Its formulation is given as follows:

(11) $$KI\left(f_{i},f_{j}\right)=\frac{\left|f_{i}\cap f_{j}\right|-\left(\left|f_{i}\right|\times \left|f_{j}\right|\right)/N}{min\left(\left|f_{i}\right|,\left|f_{j}\right|\right)-\left(\left|f_{i}\right|\times \left|f_{j}\right|\right)/N}$$

In the above expression, the similarity metric takes values in the range [−1, 1]. A value closer to 1 indicates a higher similarity between the two feature subsets. When the index equals 1, the two subsets are identical. When the value approaches 0 or becomes negative, it suggests a low similarity between the feature subsets, indicating relatively poor stability of the feature-selection algorithm.

Let $\left|f_{i}\cap f_{j}\right|$ denote the number of common features between subsets $f_{i}$ and $f_{j}$, while $\left|f_{i}\right|$ and $\left|f_{j}\right|$ represent the number of features in subsets $f_{i}$ and $f_{j}$, respectively. N denotes the total number of available features.

The average Kuncheva index is calculated as the mean of the similarity indices over all feature subsets, providing an overall evaluation of algorithm stability. It is defined as follows:

(12) $$AS=\frac{2}{n\left(n-1\right)}\sum_{i=1}^{n-1}\sum_{j=i+1}^{n}KI\left(f_{i},f_{j}\right)$$

Among them, $KI\left(f_{i},f_{j}\right)$ are the Kuncheva indices of $f_{i}$ and $f_{j}$, n indicating the number of cross-validations.

To ensure fair comparison among different methods, the Kuncheva index is calculated for each feature-selection algorithm under an identical number of selected features, and the average value is used as the stability metric, as reported in Table 4.

As shown in Table 4, significant differences in stability can be observed among the compared methods under a fixed number of selected features. RCMF consistently achieves higher stability scores across multiple feature subset sizes, with superior average stability compared to other methods, demonstrating its robustness under data perturbations.

### 3.5. Correlation Analysis of Evaluation Metrics

To validate the rationality of the proposed multi-metric fusion evaluation framework, this paper analyzes the relationships among the residual-based conditional discriminative measure, predictive contribution, and stability score. Specifically, a statistical correlation analysis of these metrics across different datasets is conducted to evaluate the degree of information overlap and their complementarity. In the proposed method, the final score is jointly determined by these three types of metrics. Although each metric characterizes feature importance from a different perspective, the predictive contribution is derived from a strongly nonlinear model, and its importance scores may partially overlap with the conditional discriminative information. Therefore, it is necessary to quantitatively analyze the relationships among these metrics to verify whether they provide complementary information rather than simple redundancy.

First, the three types of scores—residual-based conditional discriminative measure, predictive contribution, and stability score—are computed for all candidate features. Based on these scores, the Spearman rank correlation coefficient is adopted to measure the correlation between different metrics, thereby evaluating their consistency in feature ranking. The formulation is given as follows:

(13) $$\rho =1-\frac{6\sum d_{i}^{2}}{n(n^{2}-1)}$$

where ρ denotes the Spearman rank correlation coefficient between two evaluation metrics, $d_{i}$ represents the difference in the ranking of the i-th feature under the two metrics, and n denotes the total number of features. A larger value of ρ indicates a higher degree of consistency between the two metrics in feature ranking, whereas a smaller value suggests that the two metrics exhibit noticeable differences in evaluating feature importance.

Furthermore, to evaluate the consistency of different metrics in selecting high-scoring features, the top k features are selected according to each metric, and the overlap ratio between the resulting feature subsets is calculated. The formulation is given as follows.

(14) $$Overlap=\frac{\left|S_{1}\cap S_{2}\right|}{k}$$

where $S_{1}$ and $S_{2}$ denote the sets of the top k features selected by two metrics, respectively, and $S_{1}\cap S_{2}$ represents the number of common features shared by the two sets. A larger value of this metric indicates stronger consistency between the two metrics in selecting high-scoring features, whereas a smaller value suggests that the two metrics tend to identify important features from different perspectives.

To further analyze the correlations among different evaluation metrics and their consistency in selecting high-scoring features, three representative datasets—Zoo, IoT-23 Cleaned, and IoT Device Identification—are selected for experimental validation.

First, for all candidate features, the residual-based conditional discriminative measure (RCMI), predictive contribution (PC), and stability score (S) are computed. The Spearman rank correlation coefficient is then used to perform a statistical analysis of the correlations among these metrics, with the results presented in Table 5.

Based on this, to further evaluate the consistency of different metrics in selecting high-scoring features, the top *k* features are selected according to each metric, and the number of overlapping features and the overlap ratio between different feature subsets are calculated. The corresponding results are shown in Table 6.

As shown in Table 5, the correlations among different evaluation metrics are generally at a moderate to low level. Specifically, the correlation coefficients between RCMI and PC on the three datasets are 0.60, 0.53, and 0.45, respectively, indicating a certain degree of consistency in feature ranking, although the correlation is not strong. The correlations between RCMI and the stability score (S) are relatively low, with values of 0.28, 0.24, and 0.19, suggesting that these two metrics differ significantly in their evaluation perspectives. The correlation between PC and S falls between the two, but remains at a relatively low level overall.

As the feature dimensionality of the datasets increases, the correlations among the metrics exhibit a decreasing trend. This indicates that in high-dimensional feature spaces, different evaluation metrics capture feature importance in increasingly distinct ways.

Further observations from Table 6 reveal that the consistency among different metrics in selecting high-scoring features is also relatively low. On the Zoo dataset, the overlap ratios of feature subsets range from 0.25 to 0.375. On the IoT-23 Cleaned dataset, the overlap ratios decrease to the range of 0.20 to 0.333. On the IoT Device Identification dataset, the overlap is the lowest, ranging only from 0.10 to 0.22.

These results indicate that different metrics tend to identify important features from different perspectives, leading to noticeable differences in the selected high-scoring feature subsets. This effect becomes more pronounced in high-dimensional datasets, suggesting that a single metric is insufficient to fully capture feature importance.

The above findings further demonstrate that although predictive contribution, derived from a strongly nonlinear model, is capable of capturing complex feature relationships, its importance scores do not fully encompass the discriminative information modeled through conditional dependency. RCDM, predictive contribution, and stability score evaluate features from three complementary perspectives—conditional discriminative information, model-driven contribution, and robustness. While there exists a certain degree of correlation among them, they are not redundant and provide complementary information. This validates the rationality of the proposed multi-metric fusion strategy.

### 3.6. Ablation Study

Accuracy reflects the overall proportion of correctly classified samples. However, in scenarios with class imbalance or high sensitivity to misclassification, a single evaluation metric may not fully characterize model performance. To comprehensively evaluate the classification performance of the model after ablation, the F1-score is further introduced as an additional evaluation metric.

F1-score is a performance evaluation metric widely used in machine learning, particularly for classification tasks [35]. It is defined as the harmonic mean of precision and recall, providing a balanced assessment of model performance. The calculation formula is given as follows:

(15) $$\begin{array}{c} F1=2\times \frac{Precision\times Recall}{Precision+Recall}=2\times \left(\frac{TP^{2}}{\left(TP+FP\right)\left(TP+FN\right)}\right)/\left(\frac{TP}{TP+FN}+\frac{TP}{TP+FN}\right)\\ =\frac{2TP}{2TP+FP+FN} \end{array}$$

The F1-score provides a balanced measure of precision and recall, making it particularly suitable for imbalanced datasets. A higher F1-score indicates a better trade-off between these two metrics. Its value ranges from 0 to 1, where 1 represents the best performance and 0 represents the worst.

To further validate the effectiveness of each component in the proposed RCMF algorithm, ablation experiments are conducted to analyze the impact of different modules on overall performance. Using the IoT-23 Cleaned dataset, several variant models are constructed by removing the residual-based conditional discriminative measure (RCDM), predictive contribution (PC), stability score (S), and the feedback mechanism (F), respectively. In addition, while keeping other components unchanged, RCDM is replaced with traditional mutual information (MI) to construct a comparative model, aiming to evaluate the role of each module in the feature-selection process. By conducting a comprehensive comparison with the full model, the contribution of each component to the overall performance of the algorithm can be systematically assessed. The experimental results are presented in Table 7.

According to the experimental results in Table 7, each key component has a different impact on the overall performance of the RCMF algorithm. When the residual-based conditional discriminative measure (RCDM) is removed, the classification accuracy drops significantly from 97.56% to 90.56%, the F1-score decreases from 0.95 to 0.88, and the Kuncheva index also declines, indicating the most severe performance degradation. This demonstrates that incorporating conditional dependency information in feature evaluation plays a crucial role in enhancing the discriminative capability of the selected feature subset.

When the predictive contribution (PC) module is removed, the accuracy decreases to 93.23% and the F1-score to 0.92, accompanied by a decline in stability. This suggests that model-driven feature importance can effectively reflect the actual contribution of features to the classification task, thereby improving feature-selection performance. When the stability evaluation mechanism is removed, the classification performance shows only a slight decrease, while the Kuncheva index drops significantly to 0.72. This indicates that the primary role of this module is to improve the consistency of feature-selection results under different data partitions, with relatively limited direct impact on classification performance. When the feedback mechanism is removed, the accuracy and F1-score decrease to 94.28% and 0.93, respectively, and the Kuncheva index becomes 0.85. This suggests that the dynamic update process based on the current feature subset contributes to optimizing the feature-selection results to a certain extent. Furthermore, replacing RCDM with traditional mutual information (MI) results in noticeably inferior performance compared to the full model, indicating that the proposed RCDM has advantages in capturing conditional discriminative information.

Overall, the complete RCMF model achieves superior performance in both classification accuracy and stability metrics. The experimental results demonstrate that the proposed modules work collaboratively during the feature-selection process, jointly improving the overall performance of the algorithm.

## 4. Discussion

### 4.1. Overall Performance and Ablation Summary

This study aims to develop a feature-selection method that balances classification performance, feature compression capability, and selection stability in high-dimensional multi-source feature scenarios, while systematically analyzing the roles of the residual-based conditional mutual information module, predictive contribution module, stability evaluation mechanism, and feedback optimization mechanism within the overall framework.

Under a unified experimental setting, comparisons with IWFS, RF, RFECV, and CRFS demonstrate that RCMF achieves competitive average classification accuracy on most datasets. Meanwhile, the corresponding standard deviations are generally lower, indicating that the proposed method exhibits strong stability under different data partitions. Moreover, while maintaining high recognition performance, RCMF effectively reduces the number of selected features, suggesting that the proposed composite scoring mechanism can both eliminate redundant features and enhance the discriminative capability of the selected feature subset.

The ablation study further validates the contribution of each component. When the residual-based conditional mutual information module is removed, both classification performance and stability decrease, highlighting the importance of conditional dependency modeling in feature evaluation. Removing the predictive contribution module leads to a decline in overall performance, indicating that model-driven feature importance feedback helps align feature evaluation with the actual classification task. When the stability evaluation mechanism is excluded, the Kuncheva index drops significantly, demonstrating its critical role in ensuring the consistency of feature-selection results. Similarly, removing the feedback mechanism results in performance degradation, indicating that the iterative update process based on the current feature subset enhances the adaptability of feature weighting.

Overall, the RCMF method achieves a favorable balance among classification performance, feature compression, and selection stability. Each component plays a distinct role at different stages, and their coordinated interaction jointly optimizes the feature subset, rather than functioning as a simple aggregation.

### 4.2. Module Interaction and Synergistic Mechanism

The ablation results indicate that each component performs a distinct function in the feature-selection process and continuously contributes to the overall performance. The residual-based conditional mutual information module captures the incremental information of features under the constraints of conditional variables. Its introduction extends feature evaluation from simple correlation analysis to modeling conditional dependencies, thereby enhancing the independent discriminative capability of features within a multi-source feature context. The predictive contribution module incorporates a model-driven evaluation mechanism, linking feature scoring with the actual classification task and making the selection process more aligned with the final optimization objective. The stability mechanism, based on a bootstrap sampling strategy, quantifies the consistency of feature selection across different data subsets, thereby improving the robustness and reliability of the selected feature subset. The feedback mechanism performs iterative updates based on the current feature subset, dynamically adjusting feature weights and enabling adaptive optimization during the selection process.

Experimental results demonstrate that the complete RCMF method achieves superior performance in both classification metrics and stability metrics. Compared with ablated variants in which any individual module is removed, the full model exhibits a better balance between performance and stability, indicating that the components operate collaboratively at different stages. Specifically, residual-based conditional modeling provides fundamental discriminative information, the model-driven feedback mechanism further reinforces effective features, and the stability evaluation constrains the consistency of the selection results. The combined effect of these mechanisms enables the feature-selection process to enhance discriminative capability while maintaining stability, thereby improving the overall applicability of the method under diverse data conditions.

### 4.3. Practical Implications and Computational Analysis

From an application perspective, high-dimensional multi-source feature environments impose higher requirements on the discriminative capability, stability, and scalability of feature-selection methods. In practical IoT scenarios, data sources are diverse and feature dimensions are high, with redundancy and potential interference commonly existing among features from different sources. While maintaining high classification performance, RCMF effectively reduces feature dimensionality, thereby decreasing the input size without compromising recognition accuracy. This property makes it suitable for application scenarios that require a balance among model complexity, inference efficiency, and result stability, such as device identification and security monitoring tasks.

In terms of computational aspects, the proposed method mainly involves conditional dependency modeling, model-driven feature contribution evaluation, and stability analysis. Compared with traditional filter-based methods, RCMF introduces model training and iterative evaluation processes, leading to increased computational cost. However, under the current experimental scale, the overall complexity remains within an acceptable range. Moreover, the method exhibits a modular structure, which facilitates parallel computation and process optimization during implementation. Future work may incorporate distributed computing strategies, feature pre-screening mechanisms, and model lightweight design to further improve computational efficiency, making the method applicable to larger-scale datasets or real-time scenarios.

In addition, the current experiments are conducted on multiple public datasets covering different scales and data characteristics. To further enhance the applicability of the proposed method, future research may extend the evaluation to cross-domain data scenarios and more complex real-world environments, such as conditions involving distribution shifts, thereby enabling a more comprehensive assessment of its transferability and generalization performance under varying data distributions.

### 4.4. Limitations and Future Work

Although experimental results on multiple public datasets demonstrate that RCMF achieves strong performance in both classification accuracy and stability, several limitations remain. First, the residual-based conditional discriminative measure relies on a residual approximation derived from linear regression to estimate the incremental information of features under the constraints of conditional features. In highly nonlinear scenarios or in the presence of complex interactions, this approximation may not fully capture the true conditional dependencies, thereby affecting the accuracy of the estimated feature contributions. How to more accurately model conditional incremental information while maintaining computational feasibility remains an important direction for future research.

Second, the feedback optimization mechanism involves multiple rounds of model training and feature re-evaluation. While the computational cost is acceptable for moderately sized datasets, it may become a bottleneck in large-scale data scenarios or environments with frequent updates. Future work may focus on improving the iteration strategy, reducing redundant training cycles, adopting incremental update mechanisms, or leveraging parallel computation to reduce the overall computational burden.

Future research directions may include:

- Improving conditional dependency estimation methods to better capture complex nonlinear relationships;
- Optimizing the feedback weight update strategy to improve convergence efficiency and computational performance;
- Conducting cross-dataset and cross-scenario validation to systematically evaluate generalization capability;
- Developing lightweight implementations to improve applicability in resource-constrained environments;
- Providing more rigorous theoretical analysis, including formal proofs of convergence and stability.

These directions will help further improve the reliability and applicability of the proposed method in more complex real-world scenarios.

## 5. Conclusions

This chapter addresses the problem of feature selection in high-dimensional multi-source IoT data and proposes a feature-selection method based on residual-approximate conditional mutual information and a feedback fusion mechanism. The method constructs a feature-evaluation framework from three aspects: conditional information modeling, model-driven feature contribution assessment, and stability analysis. Feature importance is obtained through weighted fusion, and an iterative feedback mechanism is introduced to update feature weights dynamically. By introducing a residual-based approximation, the evaluation of high-dimensional conditional dependencies is transformed into residual-level correlation analysis, which helps reduce computational complexity while improving the stability of feature evaluation. In addition, a bootstrap-based stability assessment is incorporated to enhance the consistency of feature-selection results under different data partitions.

Experimental results on multiple public datasets show that the proposed method achieves stable performance in terms of classification accuracy, feature compression, and selection stability. On most datasets, RCMF is able to reduce the number of selected features while maintaining strong recognition performance, indicating its effectiveness in both discriminative capability and dimensionality reduction. Ablation studies further illustrate the roles of different components, showing that each module contributes at different stages and jointly improves overall performance.

It should be noted that the proposed method involves multiple rounds of model training and stability evaluation, resulting in higher computational cost compared with simple statistical approaches. In large-scale or real-time application scenarios, computational efficiency may become a limiting factor. Future work could focus on reducing computational overhead through feature pre-screening, model lightweight design, and parallel computation. In addition, incorporating incremental update strategies may help the method better adapt to dynamic data environments.

Overall, the proposed method achieves a reasonable balance among classification performance, feature compression, and stability, and shows potential for application in complex multi-source data scenarios. Further work is needed to improve efficiency and facilitate practical deployment in real-world IoT environments.

## Figures and Tables

**Table 1:** Dataset.

ID	Name	Features	Classes	Samples
1	Zoo	16	7	101
2	Car Evaluation	6	4	1728
3	Seeds	7	3	210
4	Raisin	7	2	900
5	IoT-23 Cleaned	36	3	1597
6	TON_IoT	43	10	211,043
7	RT-IoT2022	83	12	123,117
8	IoT Device Identification	297	10	1000

**Table 2:** The highest classification accuracy of 5 algorithms on each data set.

ID	IWFS	RF	RFECV	CRFS	RCMF
1	94.07 ± 0.21	96.93 ± 0.25	96.73 ± 0.19	95.19 ± 0.20	98.03 ± 0.15
2	95.60 ± 0.23	97.80 ± 0.17	95.70 ± 0.22	95.60 ± 0.21	99.54 ± 0.13
3	90.86 ± 0.25	94.76 ± 0.20	92.86 ± 0.24	94.86 ± 0.26	96.76 ± 0.17
4	86.20 ± 0.29	85.80 ± 0.26	85.51 ± 0.27	86.78 ± 0.23	87.28 ± 0.20
5	97.49 ± 0.18	97.56 ± 0.16	97.56 ± 0.16	97.62 ± 0.15	97.56 ± 0.16
6	99.22 ± 0.13	99.73 ± 0.10	99.70 ± 0.11	99.32 ± 0.12	99.82 ± 0.09
7	99.79 ± 0.09	99.77 ± 0.10	99.80 ± 0.09	99.81 ± 0.08	99.83 ± 0.08
8	82.60 ± 0.27	83.40 ± 0.25	82.40 ± 0.28	83.40 ± 0.25	84.20 ± 0.22

**Table 3:** The number of selected features at the highest recognition rate of the RCMF algorithm and other algorithms on each dataset.

ID	Total Features	IWFS	RF	RFECV	CRFS	RCMF
1	16	13	12	11	11	9
2	6	5	6	6	6	6
3	7	6	6	7	7	6
4	7	7	7	7	3	3
5	36	30	18	23	34	34
6	43	13	15	15	32	13
7	83	57	82	53	57	57
8	297	80	10	20	250	30
Mean	61.88	26.38	19.5	17.75	50	19.75

**Table 4:** The stability results of each feature-selection algorithm with different number of features.

Features	IWFS	RF	RFECV	CRFS	RCMF
1	0.89	1.00	1.00	0.72	1.00
2	0.68	0.97	0.92	0.68	0.98
3	0.82	0.91	0.91	0.64	0.94
4	0.78	0.88	0.94	0.56	0.88
5	1.00	0.94	0.85	0.66	0.97
6	0.84	0.86	0.87	0.74	0.89
7	0.85	0.92	0.95	0.73	0.92
Mean	0.83	0.92	0.92	0.67	0.94

**Table 5:** Spearman Rank Correlation Coefficients among Evaluation Metrics across Datasets.

Indicator Pairs	Zoo	IoT-23 Cleaned	IoT Device Identification
RCMI & PC	0.60	0.53	0.45
RCMI & S	0.28	0.24	0.19
PC & S	0.41	0.35	0.29

**Table 6:** Feature Set Overlap Ratios among Evaluation Metrics across Datasets.

Indicator Pairs	Zoo (*k* = 8)		IoT-23 Cleaned (*k* = 15)		IoT Device Identification (*k* = 50)	
	Overlapping Features	Overlap Ratio	Overlapping Features	Overlap Ratio	Overlapping Features	Overlap Ratio
RCMI & PC	3	0.375	5	0.333	11	0.220
RCMI & S	2	0.250	3	0.200	5	0.100
PC & S	3	0.375	4	0.267	6	0.120

**Table 7:** The Results of the Ablation Experiment.

Algorithm	Accuracy	F1	Kuncheva Index
w/o RCMI	90.56	0.88	0.84
w/o PC	93.23	0.92	0.87
w/o S	95.11	0.94	0.72
w/o F	94.28	0.93	0.85
RCMF (RCDM-MI)	92.35	0.90	0.82
RCMF	97.56	0.95	0.91

## Data Availability

The data used in this study are publicly available from the UCI Machine Learning Repository http://archive.ics.uci.edu/ml/index.php (accessed on 10 August 2025) and Kaggle https://www.kaggle.com/datasets (accessed on 20 August 2025). The specific datasets are cited in the manuscript.
